# Production of secondary particles from cosmic ray interactions in the earth’s atmosphere: Implications for annual effective dose, ^14^C/^12^C ratio, and magnetic field effects

**DOI:** 10.1371/journal.pone.0328915

**Published:** 2025-10-23

**Authors:** Mehdi Hassanpour, Mohammadreza Rezaie, Yassin Heydarizade, Mohammad Rashed Iqbal Faruque, Marzieh Hassanpour

**Affiliations:** 1 Space Science Centre (ANGKASA), Institute of Climate Change (IPI), Universiti Kebangsaan Malaysia, Bangi, Selangor D. E., Malaysia; 2 Department of Nuclear Engineering, Faculty of Modern Sciences and Technologies, Graduate University of Advanced Technology, Kerman, Iran; Universiti Teknologi Malaysia, MALAYSIA

## Abstract

This study investigates the interaction of cosmic rays with Earth’s atmosphere, focusing on the production of secondary particles, electromagnetic waves, and their dosimetric implications. Using the Geant4 toolkit, the deviation of cosmic rays due to Earth’s magnetic field, the annual effective dose of secondary particles, and the ^14^C/^12^C isotopic ratio were calculated. The results demonstrate that electromagnetic waves generated in the atmosphere exhibit an energy spectrum ranging from 0 to 500 MeV. The estimated annual effective dose at ground level is 2.28E-06 mSv/y, while the dose from secondary protons in a human-equivalent phantom is 0.105 mSv/y, approximately 30% of the total dose from cosmic rays (0.33 mSv/y). Analysis of magnetic field effects reveals that heavier particles, such as iron and nickel, experience greater deviations in their trajectories compared to lighter elements like protons and oxygen. Furthermore, the initial ^14^C/^12^C ratio in the upper atmosphere was calculated as 0.119, which decreases to 1.2E-12 at ground level due to atmospheric mixing and chemical interactions. These findings highlight the significance of cosmic ray interactions in atmospheric ionization, isotopic composition, and radiation dose estimations.

## Introduction

High-energy cosmic rays exist outside the Earth’s atmosphere [1–3]. These rays are predominantly composed of hydrogen ions (protons), helium nuclei (alpha particles), and heavier ions, with energies ranging from several MeV to several GeV and even extending to the TeV scale [4,5]. When these high-energy particles enter the Earth’s atmosphere, they primarily interact with light nuclei such as nitrogen and oxygen, initiating various nuclear reactions. One of the key processes involved is spallation (Spallation) [6,7]. Spallation is a high-energy nuclear reaction in which a cosmic ray particle (usually a proton or an alpha particle) collides with a target nucleus, causing the emission of several nucleons or lighter clusters. As a result, an excited residual nucleus is formed, which may decay into unstable or rare isotopes [8]. The study of spallation interactions in the Earth’s atmosphere dates back to the mid-20th century when the presence of specific isotopes like ^14^C and 10Be was detected in sedimentary samples and polar ice [9]. These discoveries led to the development of a theory whereby cosmic rays interact with atmospheric nuclei to produce lighter nuclei through spallation [10,11]. In these interactions, compound nuclei are formed, which typically de-excite via neutron or gamma-ray emission, fission-like processes, or particle evaporation [12,13]. In addition to the rare isotopes produced, secondary particles such as neutrons, pions, muons, and gamma photons are also generated in large quantities [14,15]. These secondary products not only play a role in the generation of cosmic radiation in the atmosphere but also have applications in areas such as dosimetry, modeling of Earth’s magnetic field effects, and isotopic analysis.

Understanding these interactions is essential for evaluating the radiation dose experienced at various altitudes and environments. Recent advancements in simulation codes such as Geant4 toolkits have significantly enhanced the precision of spallation process modelling [16,17]. These tools provide accurate simulations of nuclear interactions and the analysis of the composition and density of secondary products. Consequently, spallation is recognized as one of the most important processes for the generation of secondary radiation in the Earth’s atmosphere.

Several studies have investigated these effects, providing insights into radiation exposure from cosmic rays and their secondary by-products under different conditions. Vuković et al. [14] evaluated the dose resulting from secondary particle and proton interactions at various aircraft altitudes. Their study reported that the annual occupational effective dose for aircraft crew working 500 hours per year was approximately 1.64 mSv, which is below the annual dose limit of 6 mSv. They also highlighted that at flight altitudes, the dose equivalent primarily consists of neutrons (55%), followed by electrons and positrons (20%), protons (15%), and photons and muons (5%). Goldhagen et al. [15] focused on the cosmic ray dose received on the Earth’s surface and at aviation altitudes. They reported that neutrons induced by cosmic rays contribute approximately half of the dose equivalent at aviation altitudes. At 20 km altitude, the effective dose rate due to neutrons was measured at 5.8 mSv/h, showing significant variation with geomagnetic latitude. Astronauts on the Moon are continuously exposed to galactic cosmic rays (GCRs) and their secondary by-products, such as gamma rays and neutrons. Hayatsu et al. [18] estimated the annual ambient dose equivalent on the lunar surface to be about 570 mSv during periods of intermediate solar activity, with neutrons contributing 50 mSv of the total dose. This emphasizes the need to account for neutron exposure in future lunar missions. Additionally, Chen et al. [19] assessed the cosmic ray dose received by astronauts during space missions and highlighted those primary heavy ions, such as iron nuclei, and secondary particles like neutrons significantly influence the dose. Their findings suggest that mission trajectory and duration critically impact the total radiation dose experienced by astronauts.

According to the NCRP report (2009), the estimated annual effective dose of cosmic rays on Earth’s surface is approximately 0.33 mSv/y. This study specifically explores the secondary protons produced in Earth’s atmosphere through the spallation of cosmic protons, as well as oxygen, iron, and nickel particles [20]. The interaction of electromagnetic waves and heavy particles within the Earth’s atmosphere generates additional secondary particles, which can further contribute to radiation exposure on Earth’s surface, alongside radiation from other sources.

While extensive research has been conducted on the effects of various cosmic rays on Earth, the amount of change in these rays in the main layers of the atmosphere, as well as the quantity of secondary radiation from these particles, has not been estimated. To calculate the dose caused by secondary cosmic rays, the spectrum of cosmic rays is first extracted from various scientific sources. Then, the number of electromagnetic waves and secondary produced particles, the ratio of ^14^C/^12^C, and changes in the radius of rotation of particles based on Earth’s magnetic field are examined using the Geant4 toolkit [21].

The ratio of carbon-14 to carbon-12 is a quantity commonly used for dating plant and animal fossils [22]. Carbon-14 is produced in the Earth’s atmosphere as a result of interactions with cosmic rays [23]. When a living organism, whether plant or animal, dies, its connection to the carbon in the air and the production of carbon in its tissues ceases. Over time, the ratio of carbon-14 to carbon-12 decreases due to the decay of carbon-14. By measuring the extent of this decrease, the sample’s age can be determined.

The aim of this study is to investigate the interaction of cosmic rays with Earth’s atmosphere and to evaluate the resulting secondary radiation. Specifically, the study aims to (1) estimate the annual effective dose from secondary protons and gamma rays produced through cosmic ray interactions with atmospheric elements, (2) assess the deviation and curvature of cosmic ray trajectories caused by Earth’s magnetic field, and (3) determine the ^14^C/^12^C isotopic ratio in the upper atmosphere resulting from cosmic ray spallation. The procedure for completing this task is outlined below.

## Materials and methods

### Description of the Geant4 toolkit program

Monte Carlo simulations are widely recognized for their capability to model particle movements within a target material and predict interaction outcomes based on probabilistic methods. This approach provides a robust and efficient tool for investigating complex nuclear processes and reactions [24–27].

**Fig 1:**
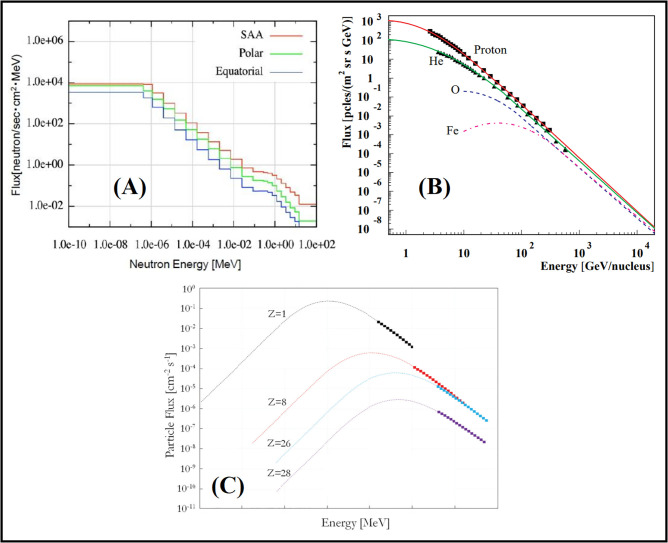
The energy spectrum of cosmic rays: (A) Cosmic neutrons [28] (Open Access), (B) Alpha particles [29] (APS License Number: RNP/24/DEC/086458), (C) Proton, Oxygen, Iron, and Nickel Cosmic Rays [30] (Elsevier Permission License Number: 5926970238868).

This study conducted simulations using the Geant4 code, a Monte Carlo-based simulation platform designed for modeling particle interactions with matter. The input file for the code included the definition of the system geometry, specifications of the particle source, selection of physical models for nuclear and electromagnetic interactions, and configurations related to detectors and data recording parameters. Material compositions were precisely defined in this file, and for each component, physical parameters such as density and atomic number were specified. Additionally, boundary conditions and simulation processes were configured to ensure maximum accuracy in replicating particle behavior. The energy spectrum of cosmic rays, shown in Fig 1, is first selected as the radiation source to assess the radiation dose induced by the entry of cosmic rays into the Earth’s magnetic field.

As illustrated in Fig 1, the primary components of cosmic rays include protons, alpha particles, and heavier nuclei such as oxygen, iron, and nickel. These particles exhibit a wide energy spectrum, typically ranging from a few MeV up to 10⁷ MeV, while secondary neutrons produced through interactions span energies up to 10² MeV. These cosmic rays are characterized as radiation sources in the Geant4 toolkit based on their energy and probability. In the macro file, these energy spectra are specified as GPS sources. The atmosphere (U.S. Standard Atmosphere [31]) and the human phantom are defined in the Detector Construction library. The Earth’s magnetic field, set at 10^−5^ T in this library, is also considered. Secondary particles generated in the atmosphere by each type of cosmic ray can be obtained using the Geant4 code outputs specified in the RUNManager library. The energy spectrum of electromagnetic waves or photons emitted by the atmosphere is also calculated. Depending on the application, various physics lists can be utilized in the Geant4 toolkit.

The applicability of a given physics list is determined by numerous factors, including the type of incident particle, beam energy range, and required precision. For Geant4 version 10.1, three physics lists were examined: QGSP-BERT-HP, QGSP-BIC-HP, and QGSP-BIC-AllHP selected for their suitability in low-energy applications such as medical and radiological protection. In this study, the QGSP-BERT-HP physics list was employed, which is known for its capability to accurately model secondary particles produced by spallation interactions.

**Fig 2:**
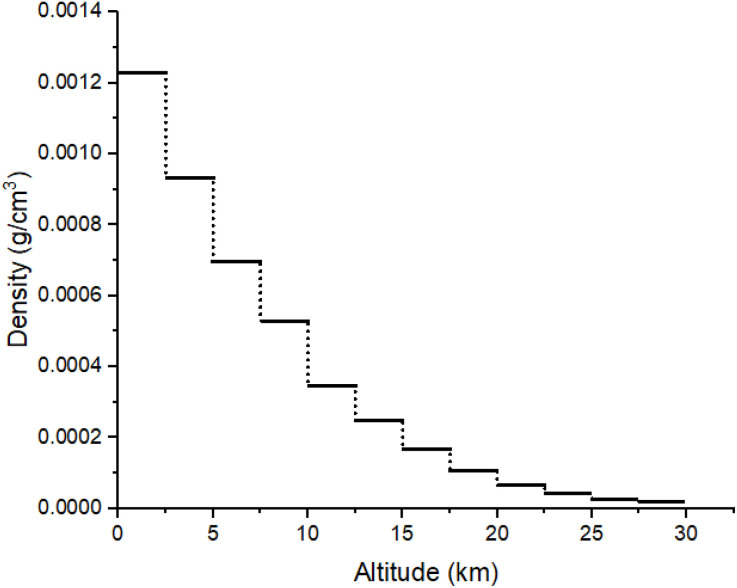
Air density variation with altitude based on U.S. Standard Atmosphere data [31].

The simulated air phantom has dimensions of 100 × 100 × 30000 m³. The air density varies at different altitudes, and the data were obtained from the ATMOSPHERE PROPERTIES website according to altitude [31]. The corresponding plot is shown in Fig 2. The material definition for air was also adopted from the Geant4 materials library as G4_AIR. Table 1 displays the percentage composition of air elements in the Geant4 toolkit.

**Table 1:** The percentage of air elements in the atmosphere based on the Geant4 toolkit.

Element	Percentage
Carbon	0.000124
Nitrogen	0.755268
Oxygen	0.231781
Argon	0.012827

**Fig 3:**
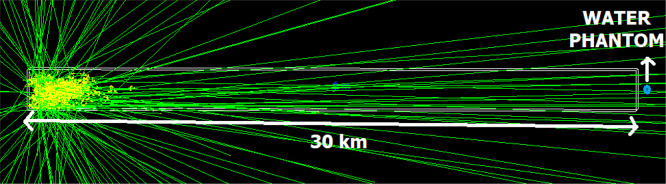
A picture of cosmic rays entering the Earth’s atmosphere and a human-equivalent phantom 30 km away from the entrance location (The scale of the figure has been adjusted from 1/30 to 5/17 for a better understanding of the scale).

Cosmic rays serve as the primary source of atmospheric ionization, playing a crucial role in determining the electrical conductivity of the atmosphere at altitudes up to 35 km [32]. As shown in Fig 3 from the Geant4 toolkit, the cosmic ray source is positioned 30 km outside Earth’s atmosphere, with radiation exposure occurring over a 30 km distance. The material definition for air was adopted from the Geant4 materials library as G4_AIR. Following the U.S. Standard Atmosphere model, the altitude from 0 to 30 km above the Earth’s surface has been divided into twelve distinct layers, each modeled with its corresponding density, as shown in Fig 2.

The collision of cosmic rays and the generation of secondary particles are most probable in the upper layers of the atmosphere. To assess the dosimetry of secondary protons and gamma rays reaching the Earth’s surface, a cylindrical water phantom with a radius of 15 cm and a height of 150 cm is placed 30 kilometers from the source. For accuracy better than 1%, the number of primary particles in the source is set to 10^6^, as determined by the code during execution.

### Trajectory of low-energy cosmic rays in earth’s atmosphere: Magnetic field effects

**Fig 4:**
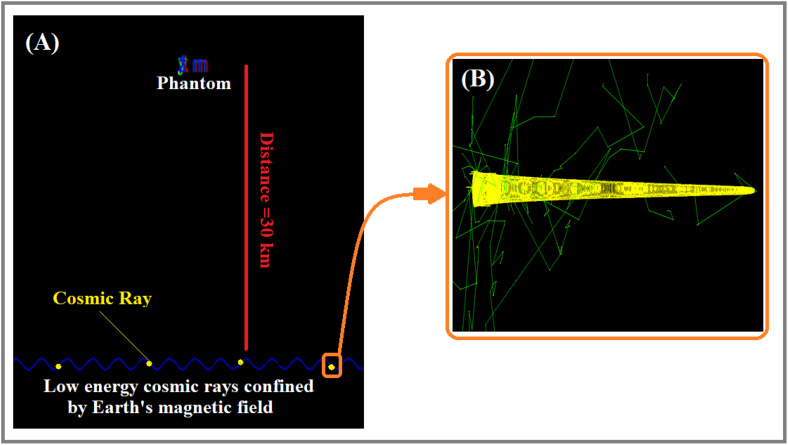
Simulation of Earth’s atmosphere under the influence of a magnetic field when low-energy cosmic rays interact with the atmosphere. (A) Trajectory of low-energy cosmic rays in Earth’s magnetic field. (B) Interaction of cosmic rays with the atmosphere.

Fig 4A and 4B illustrate the simulation model of Earth’s atmosphere and the human-equivalent phantom at a distance of 30 km. According to Fig 4A, some low-energy cosmic rays are confined within the initial layers of Earth’s atmosphere and exhibit cyclotron motion around the Earth’s magnetic field lines.

In Fig 4A, the blue sinusoidal curve represents the trajectory of low-energy cosmic ray particles trapped along the Earth’s magnetic field lines, preventing them from reaching the phantom placed at the Earth’s surface. Zooming in on the trajectory of one particle in Fig 4A yields Fig 4B, which illustrates the paths of secondary particles (shown in green) generated by the interaction of cosmic rays with the upper layers of the Earth’s atmosphere. The yellow color in Fig 4B depicts the rotational motion of a cosmic ray particle around magnetic field lines. As the particle moves forward (to the right), its energy is gradually lost, causing the radius of the circular path in which it is confined to shrink until the particle eventually comes to a stop.

**Fig 5:**
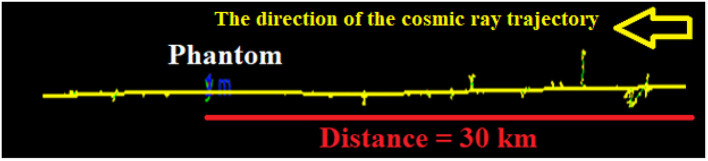
The trajectory of high-energy cosmic rays in the absence of the Earth’s magnetic field.

Fig 5 illustrates that certain high-energy cosmic rays maintain a straight trajectory when Earth’s magnetic field is neglected, thereby potentially intersecting with the phantom. As evident, along the trajectory of the cosmic ray, interactions with the Earth’s atmosphere occur, leading to the production of secondary particles, which are distributed around the path. As the particle approaches the phantom, the production of secondary particles decreases. Moreover, if the magnetic field is neglected, the possibility of the cosmic ray reaching the Earth’s surface exists.

**Fig 6:**
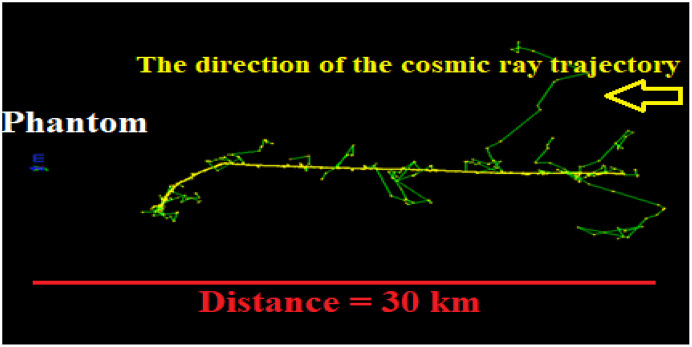
The path of high-energy cosmic rays in the presence of the Earth’s magnetic field.

In a more realistic case, the particles’ trajectories were tracked under the influence of the magnetic field. According to the findings in Fig 6, a minimal Earth’s magnetic field with a value of 1.5 × 10^−5^ *T* will affect the particle’s path, causing its deviation from a straight line towards the end of its trajectory, which potentially prevent it to reach the phantom. Additionally, the secondary particles produced under the influence of the magnetic field are deflected and scattered further from the trajectory line compared to the previous case (Fig 5).

### The trajectory of high-energy cosmic rays in the atmosphere with the effect of a magnetic field

According to the conclusions drawn from Figs 4–6, cosmic rays deviate from a straight path in the atmosphere due to the influence of Earth’s magnetic field, potentially causing them to gyrate around it. The magnetic field and force are correlated with the radius of movement. The electromagnetic force *F(N)* depends on the charge *q*(C) of cosmic particles, the Earth’s magnetic field *B*(T) with an average value of 5e-5 [33], and either the velocity *V* (m/s) or the particle’s energy *E*(J), as described by Equation 1.


F=qBV (1)

This force can trap particles within Earth’s magnetic field. Equation 2 defines the radius *r* (meter) of the cosmic ray’s rotation around Earth’s magnetic field.


$$F=qBV=\frac{mV\text{2}}{r}\;$$

$$\;\Rightarrow r=\;\frac{mV\;}{qB}$$ (2)

In Equation 2, the force $\frac{mV^{\text{2}}}{r}$ represents the centripetal force, where *m* (kg) is the mass of the cosmic ray particle. By accounting for relativistic effects on the mass of cosmic particles, Equation 2 can be reformulated based on relativistic mechanics by incorporating the Lorentz factor (γ), which represents the ratio of the moving particle’s mass to its rest mass (γ = m/m₀), resulting in the modified form presented in Equation 3.


$$r=\;\frac{mV}{qB}=\frac{\gamma m_{\text{0}}Vc}{qBc}=\frac{\text{1}}{qBc}\sqrt{{\left(T+m_{\text{0}}c^{\text{2}}\right)}^{\text{2}}-m_{\text{0}}^{\text{2}}c^{\text{4}}}=\frac{m_{\text{0}}c^{\text{2}}}{qBc}\sqrt{{\left(\frac{T+m_{\text{0}}c^{\text{2}}}{m_{\text{0}}c^{\text{2}}}\right)}^{\text{2}}-\text{1}}$$ (3)

In this equation, *m*_0_*c*^2^ represents the rest mass energy of the cosmic particle’s nucleus, and *T* denotes the particle’s kinetic energy in MeV. The rest mass energy can be expressed in terms of the particle’s mass number, while the particle’s charge can be defined based on its atomic number, as follows:


$$m_{\text{0}}c^{\text{2}}≅A\times \text{1}amu.c^{\text{2}}=\text{9}\text{3}\text{1}.\text{5}\times A[MeV]\equiv \text{9}\text{3}\text{1}.\text{5}\times {\text{1}\text{0}}^{\text{6}}\times A\times \text{1}.\text{6}\times {\text{1}\text{0}}^{-\text{1}\text{9}}[J\equiv N.m];$$

$$q=Z.e\equiv \text{1}.\text{6}\times {\text{1}\text{0}}^{-\text{1}\text{9}}Z$$

By substituting these values and assuming an average magnetic field of *B* = 5 × 10^−5^ T, Equation 3 can be further simplified and rewritten as Equation 4.


$$r=\frac{m_{\text{0}}c^{\text{2}}}{qBc}\sqrt{{\left(\frac{T+m_{\text{0}}c^{\text{2}}}{m_{\text{0}}c^{\text{2}}}\right)}^{\text{2}}-\text{1}}\equiv \frac{\text{9}\text{3}\text{1}.\text{5}\times {\text{1}\text{0}}^{\text{6}}\times A\times \text{1}.\text{6}\times {\text{1}\text{0}}^{-\text{1}\text{9}}}{\text{1}.\text{6}\times {\text{1}\text{0}}^{-\text{1}\text{9}}Z.B.c}\sqrt{{\left(\frac{T+\text{9}\text{3}\text{1}.\text{5}\times A}{\text{9}\text{3}\text{1}.\text{5}\times A}\right)}^{\text{2}}-\text{1}}$$

$$r\;(m)=\frac{\text{9}\text{3}\text{1}.\text{5}\times {\text{1}\text{0}}^{\text{6}}\times A[N.m]}{Z.\text{5}\times {\text{1}\text{0}}^{-\text{5}}\times \;\text{3}\times {\text{1}\text{0}}^{\text{9}}\;[N]}\sqrt{{\left(\frac{T+\text{9}\text{3}\text{1}.\text{5}\times A}{\text{9}\text{3}\text{1}.\text{5}\times A}\right)}^{\text{2}}-\text{1}}=\text{6}\text{2}\text{1}\text{0}\text{3}\frac{A}{Z}\sqrt{{\left(\frac{T+\text{9}\text{3}\text{1}.\text{5}\times A}{\text{9}\text{3}\text{1}.\text{5}\times A}\right)}^{\text{2}}-\text{1}\;}$$

$$\Rightarrow \text{r}\;(\text{km})=\text{62}.\text{10}\frac{\text{A}}{\text{Z}}\sqrt{{\left(\frac{\text{T}\;(\text{MeV})+\text{931}.\text{5}\times \text{A}}{\text{931}.\text{5}\times \text{A}}\right)}^{\text{2}}-\text{1}}$$ (4)

For each cosmic ray particle with a mass and atomic numbers *A* and Z, the value of *r* can be calculated using the above relation.

### Annual effective dose calculation of secondary protons and gamma rays produced by cosmic ray interaction with the atmosphere

The cylindrical water phantom with a radius of 15 cm and a height of 150 cm was selected to approximate the geometry and dimensions of an average adult human body. This is consistent with the representation of the human body as a multi-cylinder model with a spherical head, as described in [34]. The corresponding body mass for these dimensions is approximately 110 kg [35]. While this configuration may not reflect typical anthropometric characteristics in the general population, particularly in the context of radiation risk assessment, it was not intended to replicate an average human subject. Rather, it was adopted as a conservative and idealized model to simulate a worst-case exposure scenario. This modeling approach minimizes the potential underestimation of absorbed dose resulting from smaller body volumes. Moreover, in previous dosimetric studies, cylindrical phantoms with varying body masses—commonly 60, 90, and 110 kg—have been proposed to represent a range of body types [35]. In this study, only the 110 kg phantom was simulated and positioned horizontally (parallel to the ground) to maximize the effective cross-sectional area exposed to incident cosmic rays, thereby enabling the estimation of an upper-bound dose in a simplified, yet physically meaningful configuration.

The dose of cosmic rays generated in the human phantom, *D* given in Gray (Gy), can be obtained by calculating the absorbed energy *E* given in Joule (J) in the human-equivalent phantom and dividing it by the phantom’s mass (*m*), given in kg, as shown in Equation 5.


$$D=\frac{E}{m}$$ (5)

The dose of cosmic rays in the phantom can be estimated using the Run Manager library and the dose deposit (*Ddep*) function detailed in the research findings. To compute the annual effective dose, it is essential to know the flux of cosmic rays entering Earth’s atmosphere. The daily or annual effective dose of cosmic rays in the phantom can then be calculated by multiplying the flux of each cosmic ray by the probability of each cosmic particle reaching the phantom.

To calculate the total effective dose, the dose (Gy) was first determined for the phantom. Since the phantom tissue is assumed to be uniform throughout, an equivalent tissue weighting factor was assigned to each section. This value represents the average of the tissue weighting factors for 13 different organs, which, according to the ICRP 60 report [36], is given by $\overset{―}{W_{T}}=\sum \frac{W_{T}}{n=\text{1}\text{3}}=\text{0}.\text{0}\text{7}\text{7}$. As all 13 sections of the phantom, modeled as water-equivalent tissue, receive the same dose, the effective equivalent dose is calculated as the product of the particle weighting factor, the dose received, the number of tissues, and the equivalent tissue weighting factor, as given in Equation 6.


$$Annual\;effective\;dose=\;\sum W_{R}\times W_{T}\times D_{T}=\sum W_{R}\times \text{1}\text{3}\overset{―}{W_{T}}\times \;D_{T}\;\;$$ (6)

In this equation, $W_{R}$ is the radiation weighting factor, which is 1 for gamma rays, 5 for protons, and 20 for other ions (Proton, Oxygen, Iron, Nickel) [37]. $D_{T}$ refers to the dose absorbed in each tissue, equal to the dose absorbed in the entire water-equivalent phantom.

To calculate the dose (D_T_) and flux in the mentioned phantom, a cylindrical scoring mesh with the same dimensions was defined in the Macro file and positioned at the phantom’s location using the Translate command. The physical quantities of flux and dose (D_T_) were computed using the energyDeposit and cellFlux scorers, specified as/score/quantity/energyDeposit and/score/quantity/cellFlux, respectively. These quantities were saved to an output file using the/score/dumpAllQuantitiesToFile command.

### The ^14^C/^12^C ratio at the top layer of the atmosphere

The interaction of high energy cosmic rays with elements in Earth’s atmosphere, such as carbon, nitrogen, oxygen, and argon, enables the occurrence of the spallation process. This process generates secondary particles, among which the production of carbon-14 is possible. By activating the spallation process in the Geant4 toolkit and utilizing commands for recording spallation-related results in the Macro file, it becomes possible to score secondary particles based on their atomic number and mass number. Accordingly, they are recorded in the output file. Using statistical analysis in Excel, the abundance of each secondary element per 10^6^ particles for each cosmic ray is calculated. Among the secondary elements produced, the abundances of carbon-14 and carbon-12 for each cosmic ray are extracted. By dividing these abundances, the ^14^C/^12^C ratio upon cosmic ray entry into Earth’s atmosphere is determined. It is worth noting that due to the interactions of these particles with oxygen in the atmosphere, two types of gases, ^14^CO_2_ and ^12^CO_2_, are produced. Because of their dispersion in Earth’s atmosphere, the ^14^C/^12^C ratio at the surface is lower than the ratio at the time of production in the upper layers of the atmosphere.

## Results and discussion

### Results of calculating secondary particles produced by cosmic ray spallation processes with earth’s atmosphere

**Fig 7:**
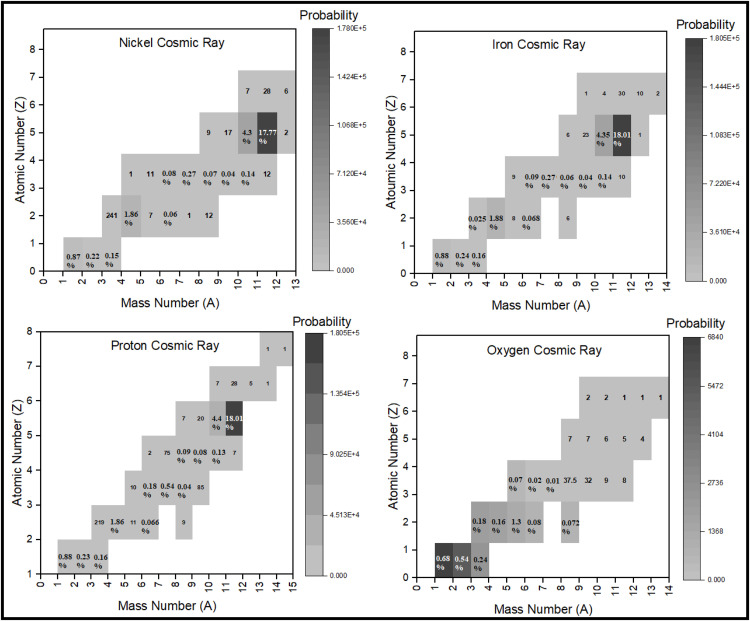
The number of cosmic ray particles produced through the interaction of protons, oxygen, iron, and nickel with the Earth’s atmosphere. Values accompanied by a percentage represent the number of particles generated per 100 incident cosmic rays, while those without a percentage indicate the number produced per one million incident cosmic rays.

By irradiating the atmosphere by a multitude of cosmic rays, secondary particles and electromagnetic waves created in Earth’s atmosphere were estimated. The energy spectra of cosmic rays (as given in Fig 1) are defined as sources in the Geant4 toolkit. Determining the elemental composition of the atmosphere is crucial for calculating the annual effective dose of secondary particles. It should be noticed here that spallation occurs as the primary/main nuclear interaction to produce secondary particles, which contribute to the annual effective dose of cosmic rays, as computed below. Fig 7 lists the secondary particles generated and the electromagnetic waves (photons) energies produced from interactions with cosmic particles.

Based on the data, the secondary particles formed consist of various components, including electrons, positrons, and other subatomic particles, as well as photons or electromagnetic waves with specific energies. Fig 7 indicates that elements like ^11^C and ^12^C are more likely generated by cosmic particles except oxygen, with a possible formation of ^14^C. It is feasible to synthesize elements with lower atomic numbers compared to cosmic oxygen. The type of cosmic particle entering the Earth’s atmosphere initially determines both the quantity and energy of the resultant radiation.

**Fig 8:**
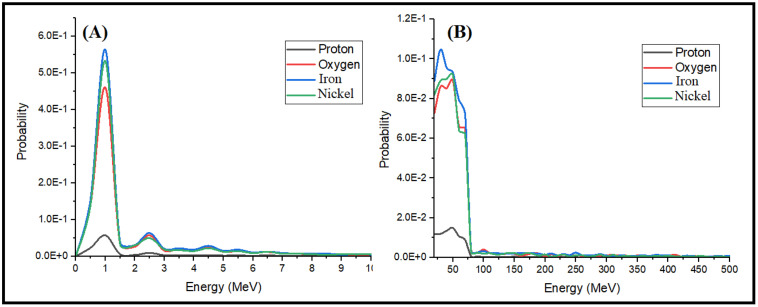
Gamma energy spectrum reaching Earth’s surface (A) less than 10 MeV, (B) greater than 10 MeV.

Based on the findings, Fig 8 illustrates the gamma spectrum released from the atmosphere due to various cosmic rays. At energies below 10 MeV, iron particles, nickel, oxygen, and eventually protons exhibit the most pronounced gamma spectra resulting from interactions with atmospheric particles. Gamma spectra are also registered at higher energies, up to 500 MeV, albeit with very low probability. Furthermore, up to an energy of 75 MeV, iron predominates in the generation of gamma rays.

### The results related to the annual effective dose

Table 2 reports the annual effective dose of electromagnetic waves and protons in a human-equivalent phantom located 30 km from the point of entry into Earth’s atmosphere for each cosmic particle. The total annual effective dose generated by cosmic rays interacting with Earth’s atmosphere is estimated by multiplying the flux of each cosmic ray by its probability. Specifically, the total annual effective dose for gamma rays and protons is 2.28E-06 mSv/year and 0.105934 mSv/year, respectively.

**Table 2:** The annual effective dose of protons and electromagnetic waves from cosmic rays that reach the human-equivalent phantom on Earth.

Primary Cosmic ray	Primary Cosmic ray Flux	Annual Effective dose of secondary particles that are produced by primary cosmic ray (mSv/year)	
		Proton	Gamma
Proton	2.75E03± 1.37E02	9.33E-02± 4.67E-03	1.21E-06± 6.05E-08
Oxygen	4.60E01± 2.30E00	1.13E-02± 5.65E-04	1.41E-07± 7.05E-09
Iron	9.91E-01± 4.95E-02	4.75E-04± 2.37E-05	3.65E-09± 1.83E-10
Nickel	5.65E-01± 2.82E-02	3.55E-04± 1.77E-05	2.28E-08± 1.14E-09
Neutron(Z = 0)	1.69E00± 8.45E-02	1.14E-12± 5.7E-14	8.92E-10± 4.46E-11
Alpha	96.03E00± 4.80E00	4.67E-04± 2.33E-05	9.05E-07± 4.53E-08
Sum=		1.06E-01± 4.70E-03	2.28E-06 ± 7.59E-08

The annual cosmic ray dose at the Earth’s surface, based on various reports and the NCRP, ranges between 0.3 to 0.33 mSv/year [20,38]. However, the results of this study indicate that 1.06E-01± 4.70E-03 mSv/year of this dose originates from secondary particles produced by the interaction of primary cosmic rays with the Earth’s atmosphere and subsequently reaching the surface. This implies that approximately 0.22 mSv/year (0.33–0.11) can be attributed to the dose from primary cosmic rays directly reaching the Earth’s surface.

### The results related to the effect of the magnetic field on the trajectory of cosmic rays in the atmosphere

Utilizing the energy spectrum data of proton, oxygen, iron, and nickel cosmic rays as shown in Fig 1, the radius of rotation of these cosmic rays is calculated using Equation 3, and the results are presented in Table 3.

**Table 3:** The radius of rotation of cosmic rays along the Earth’s magnetic field lines as a function of cosmic particle type and energy.

Cosmic Rays energy [MeV]	Curvature radius [km]					
	Proton (Z = 1, A = 1)		Alpha (Z = 2, A = 4)	Oxygen (Z = 8, A = 16)	Iron (Z = 26, A = 56)	Nickel (Z = 28, A = 59)
4.97E-01		2.03E + 00	2.03E + 00	1.01E + 00	5.84E-01	5.56E-01
1.00E + 02		2.95E + 01	2.90E + 01	1.44E + 01	8.29E + 00	7.90E + 00
5.00E + 02		7.25E + 01	6.65E + 01	3.24E + 01	1.86E + 01	1.77E + 01
1.00E + 03		1.13E + 02	9.69E + 01	4.63E + 01	2.63E + 01	2.51E + 01
5.00E + 03		3.91E + 02	2.63E + 02	1.10E + 02	5.99E + 01	5.71E + 01
1.00E + 04		7.26E + 02	4.40E + 02	1.66E + 02	8.67E + 01	8.25E + 01
5.00E + 04		3.39E + 03	1.79E + 03	5.26E + 02	2.25E + 02	2.13E + 02
1.00E + 05		6.73E + 03	3.46E + 03	9.49E + 02	3.67E + 02	3.45E + 02
5.00E + 05		3.34E + 04	1.68E + 04	4.29E + 03	1.41E + 03	1.31E + 03
1.00E + 06		6.67E + 04	3.35E + 04	8.46E + 03	2.69E + 03	2.51E + 03
4.37E + 07		2.91E + 06	1.46E + 06	3.64E + 05	1.12E + 05	1.04E + 05

**Fig 9:**
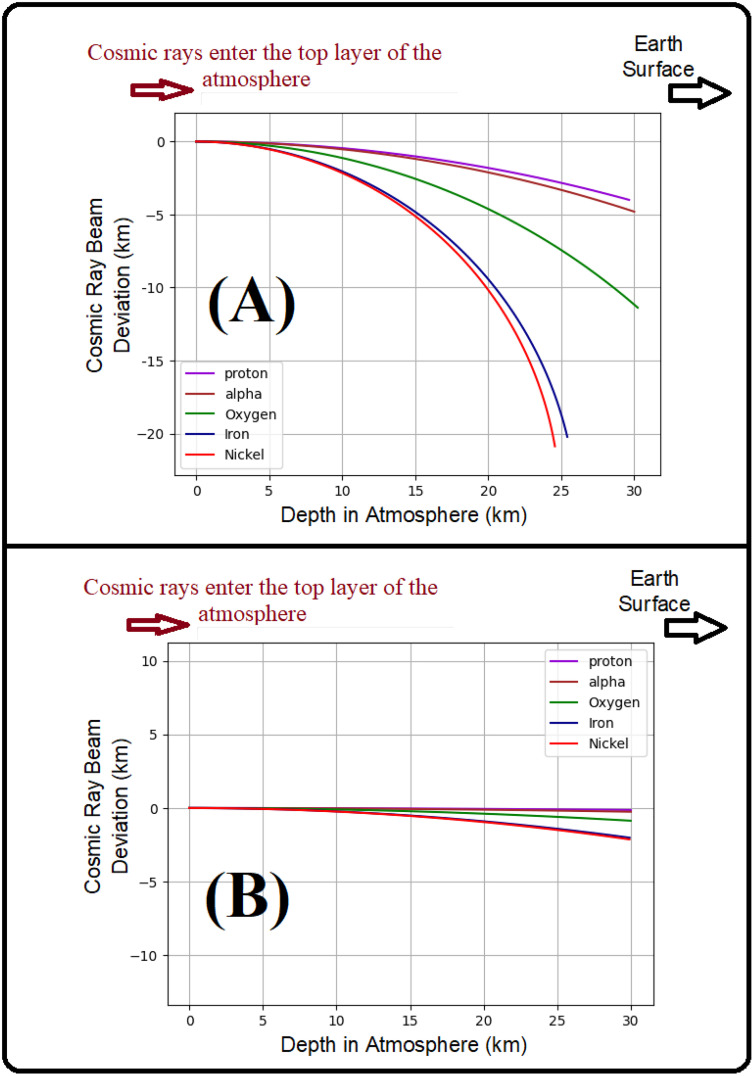
Deviation from the straight path of cosmic rays entering Earth’s atmosphere: (A) with 1 GeV energy, both with and without the influence of the magnetic field, (B) with 50 GeV energy under the influence of the magnetic field.

According to Table 3, cosmic particles experience trajectory bending due to interaction with Earth’s magnetic field, resulting in a curved or helical path. The curvature radius, presented in kilometers, depends on the particles’ energy and atomic number and characterizes the extent of magnetic deflection during atmospheric propagation. Fig 9A and 9B illustrate the minimum and maximum radii of cosmic ray trajectories entering Earth’s atmosphere with particle energies of 1 GeV and 50 GeV, respectively. These figures show that the least deviation is for protons and alpha particles, and the greatest is for iron and nickel.

It is noteworthy that a smaller deviation corresponds to a larger radius of curvature, resulting in a trajectory closer to a straight line. As shown, the deviation exhibits a direct relationship with particle mass; in other words, as the mass increases, the deviation increases. Furthermore, based on the results presented in Fig 9, it can be observed that under relativistic conditions, the deviations of alpha particles and protons are nearly identical. This indicates that the deviation is influenced not only by the mass number (A) but also by the atomic number (Z). This explains the similar curvature observed in their trajectories, a finding supported by the data in Table 3 and Fig 9.

As illustrated in Fig 9a and 9b, lighter particles exhibit larger curvature radii for equal energies and fully ionized atoms (nuclei with positive charge), resulting in less deflected trajectories. In contrast, heavier nuclei experience significantly greater deflection, preventing them from reaching the phantom located 30 km from the point of atmospheric entry. The relativistic effect is more emphasized for higher energy (50GeV), and the deviation is drastically reduced for all incoming cosmic rays, but relatively the heaviest particle beam will miss the human phantom, since it will be deviated by about 1–2 km from the incidence line. Consequently, the Fig 6 obtained as a result of GEANT-4 simulations is more consistent with the Fig 9 results. Indeed, energic particles when entering the Earth’s atmosphere will continue on almost straight line as long as their kinetic energies are comparable to their rest energy (relativistic case) and the trajectory is almost unaffected by Earth’s magnetic field, then by losing their energy due to successive collisions and interactions with air particles, they reach an energy level where the magnetic field is more significant and curvature radius is more reduced, implying a highly deviated trajectory at the end as shown in Fig 6.

### The result related to the initial ^14^C/^12^C ratio

As mentioned in previous sections, the ^14^C/^12^C ratio is one of the important parameters in dating. This section presents the results related to the calculation of the ^14^C/^12^C ratio due to cosmic ray interactions upon entering Earth’s atmosphere. Both ^14^C and ^12^C are produced when cosmic rays interact with the Earth’s atmosphere. Table 4 displays their quantities per 10^6^ cosmic rays interacting with Earth’s atmosphere.

**Table 4:** The number of ^14^C and ^12^C produced per 10^6^ cosmic rays interacting with Earth’s atmosphere.

	Proton	Oxygen	Iron	Nickel	Neutron	Alpha	Sum
^12^C	7.40E + 01 ±3.70E + 00	5.11E + 02±2.56E01	5.23E + 02±2.62E01	4.56E + 02±2.28E01	1.58E + 02±7.90E00	3.20E + 02±1.60E01	**2042** ±4.68E01
^14^C	1.40E + 01 ± 7.00E-01	6.30E + 01±3.15E00	6.10E + 01±3.05E00	6.30E + 01±3.15E00	0.00E + 00±0.00E00	4.10E + 01±2.05E00	**242** ±5.82E00

The ^14^C/^12^C ratio in the top layer of the atmosphere is 0.119 (=242/2042), as indicated by the data in Table 4. When cosmic rays interact with Earth’s atmosphere, ^14^C and ^12^C are created and mixed with oxygen to form CO_2_ gas. This gas disperses throughout the Earth’s atmosphere, causing a decrease in this ratio. Historically, CO_2_ was not predominantly of human origin. However, recent increases in human-generated CO_2_ have significantly reduced the ^14^C/^12^C ratio to approximately 1.2E-12, much lower than the initial ratio of 0.119. The current ratio of ^14^C/^12^C stands at 1.2E-12 [39]. Additionally, due to the sustained production rate of ^14^C in the upper atmosphere, the ^14^C/^12^C ratio stabilizes under current conditions after undergoing five half-lives of ^14^C (5 × 5730 years) [40].

## Conclusion

This study, using the Geant4 code, investigated the induced spallation by cosmic rays within Earth’s atmosphere, the production of secondary particles and electromagnetic waves, and their impact on the annual effective dose. The results showed that cosmic ray spallation with the atmosphere leads to the generation of secondary particles, including protons, neutrons, and gamma rays, which can potentially reach the Earth’s surface. The calculated annual effective dose for protons and secondary particles in a human-equivalent phantom was approximately 0.105 mSv/year, accounting for about 32% of the total effective dose from cosmic rays (0.33 mSv/year). Additionally, secondary gamma rays contribute with a dose of 2.28E-06 mSv/year, which is significantly lower than the safety limits defined by international standards. This study also examined the effects of Earth’s magnetic field on cosmic ray trajectories and demonstrated that low-energy particles are trapped within the magnetic field and cannot reach the Earth’s surface. In contrast, high-energy particles exhibit partial deviations from their initial paths, with the extent of deviation depending on the energy, mass, and atomic number of the cosmic particles. The calculated radii of particle deviation indicated that heavier ions, such as iron and nickel, exhibit greater deviations compared to lighter elements like protons and alpha particles. Regarding the ^14^C/^12^C ratio, the results showed that this ratio in the upper layers of the atmosphere is 0.119, which decreases to 1.2E-12 at the Earth’s surface due to atmospheric mixing and reactions with oxygen. These findings highlight the importance of cosmic ray interactions in altering the isotopic composition of the atmosphere and their implications for radiocarbon dating. This research emphasizes the need for further investigations into atmospheric modeling limitations, particularly regarding simplifications in density variations and magnetic field effects. Future studies could refine these models to incorporate atmospheric dynamics and temporal variations in cosmic ray flux. However, certain limitations in the modeling approach must be acknowledged. The simplified treatment of magnetic field effects represents a key area for future refinement. In particular, the model’s representation of the complex interactions of cosmic rays with Earth’s magnetic field could be enhanced to better reflect real-world conditions. Future studies should aim to address this limitation by incorporating a more accurate depiction of the Earth’s magnetic field and refining the model to include more detailed atmospheric dynamics, such as temporal variations in cosmic ray flux. This would lead to more precise simulations and a deeper understanding of cosmic ray behavior, improving the reliability of predictions for radiation exposure at the Earth’s surface.
